# Trends and area variations in Potentially Preventable Admissions for COPD in Spain (2002–2013): a significant decline and convergence between areas

**DOI:** 10.1186/s12913-016-1624-y

**Published:** 2016-08-09

**Authors:** Julián Librero, Berta Ibañez-Beroiz, Salvador Peiró, M. Ridao-López, Clara L. Rodríguez-Bernal, Francisco J. Gómez-Romero, Enrique Bernal-Delgado, the Spanish Atlas of Medical Practice Variation Research Group, A. Díaz-Martínez, J. A. Goicochea-Salazar, F. Rivas-Ruiz, A. Jiménez-Puente, M. M. Rodríguez del Águila, T. Molina, E. Baños, E. Angulo, E Bernal-Delgado, M. Comendeiro-Maaløe, F. R. Estupiñan-Romero, S. García-Armesto, R. Launa, N. Martínez-Lizaga, M. Ridao-López, M. Seral-Rodríguez, J. M. Abad-Diez, F. Arribas-Monzón, J. Beltrán-Peribáñez, F. Pradas-Arnal, M. Caicoya, F. Suárez, H. Sánchez-Janáriz, J. l. Alonso-Bilbao, D. Fiuza Fiuza-Pérez, G. Romero, M. Marinelli, G. Oliva, V. Ortún-Rubio, T. Salas, E. Vela, A. Sacristán-Salgado, J. García-Crespo, A. Melgosa-Arcos, L. Sangrador-Arenas, M. A. García-Sánchez, R. López-Reneo, G. Atienza-Merino, C. Carballeira-Roca, T. Queiro, M. Castro-Villares, Y. Anes del Amo, G. Montes-Salas, E. J. Castaño-Riera, M. J. Santos-Terrón, M. Zaforteza-Dezcallar, J. Ferrer-Riera, M. V. Martín-Martín, A. Cestafé, C. Bienzobas-López, R. Gómez-Lázaro, J. Palomar-Rodríguez, L. Hernando-Arizaleta, N. Álvarez-Arruti, Y. Montes-García, I. Rodrigo-Rincón, B. Ibáñez-Beroiz, F. Aizpuru, P. M. Latorre-García, A. Latorre, J. Pérez de Arriba, M. Errezola, E. Millán, C. Baixauli-Pérez, J. Librero, S. Peiró, C. L. Rodríguez-Bernal, G. Sanfelix-Gimeno, R. Meneu, R. Sotoca, J. Calabuig

**Affiliations:** 1 Centro Superior de Investigación en Salud Pública (CSISP-FISABIO), Catalunya Av. 21, 46020 Valencia, Spain; 2 Red de Investigación en Servicios de Salud en Enfermedades Crónicas (REDISSEC), Valencia, Spain; 3 NavarraBiomed - Fundación Miguel Servet, Pamplona, Spain; 4 Instituto Aragonés de Ciencias de la Salud. IIS Aragón, Zaragoza, Spain

**Keywords:** Potentially preventable hospitalizations, Chronic obstructive pulmonary disease, Small area variation analysis, Hospitalization

## Abstract

**Background:**

Potentially Preventable Hospitalizations (PPH) are hospital admissions for conditions which are preventable with timely and appropriate outpatient care being Chronic Obstructive Pulmonary Disease (COPD) admissions one of the most relevant PPH. We estimate the population age-sex standardized relative risk of admission for COPD-PPH by year and area of residence in the Spanish National Health System (sNHS) during the period 2002–2013.

**Methods:**

The study was conducted in the 203 Hospital Service Areas of the sNHS, using the 2002 to 2013 hospital admissions for a COPD-PPH condition of patients aged 20 and over. We use conventional small area variation statistics and a Bayesian hierarchical approach to model the different risk structures of dependence in both space and time.

**Results:**

COPD-PPH admissions declined from 24.5 to 15.5 per 10,000 persons-year (Men: from 40.6 to 25.1; Women: from 9.1 to 6.4). The relative risk declined from 1.19 (19 % above 2002–2013 average) in 2002 to 0.77 (30 % below average) in 2013. Both the starting point and the slope were different for the different regions. Variation among admission rates between extreme areas dropped from 6.7 times higher in 2002 to 4.6 times higher in 2013.

**Conclusions:**

COPD-PPH conditions in Spain have undergone a strong decline and a reduction in geographical variation in the last 12 years, suggesting a general improvement in health policies and health care over time. Variability among areas still remains, with a substantial room for improvement.

**Electronic supplementary material:**

The online version of this article (doi:10.1186/s12913-016-1624-y) contains supplementary material, which is available to authorized users.

## Background

Potentially Preventable Hospitalizations (PPH) are hospital admissions for certain acute illnesses or worsening chronic conditions that may be theoretically preventable with timely and appropriate outpatient care [1, 2]. PPH rely on hospital discharge data but are intended as indirect indicators of accessibility to high-quality outpatient care [3–5]. Despite a recent decline in smoking habits and age-adjusted mortality rates [6, 7], Chronic Obstructive Pulmonary Disease (COPD) remains a leading cause of morbidity and mortality [8]. Because appropriate, continuous and well-organized outpatient care in COPD patients could improve symptoms, reduce severity and avoid hospitalization, most COPD hospital admissions are incorporated into the set of PPH indicators [2], and in fact COPD hospitalizations are one of the most relevant PPH, providing between a third and a half of all cases of chronic PPHs in Spain [3, 4] and Europe [5].

Access to care and quality varies between areas and regions [9, 10]. Most PPH studies in COPD use small area variation analysis, disease mapping or other methods to show geographical variations in hospital admissions at a particular time, and/or to relate this variation to demographic or social factors, the supply of health services, physician practice styles or risk exposure [11–15]. But accessibility to high-quality healthcare can also change over time, and with a variable rhythm between areas served by different healthcare organizations. However, time trends for COPD-PPH have barely been studied and, to our knowledge, no study has simultaneously analyzed spatial and temporal variability in COPD-PPH. Understanding geographical variations and trends in COPD-PPH is important for assessing accessibility to appropriate care and also for formulating public health initiatives to reduce the burden of this disease. The objectives of this study were to estimate the population age and sex standardized relative risk (RR) of hospitalization for COPD-PPH by year and area of residence in the Spanish National Health System (sNHS) during the period 2002–2013, and to describe its average trends and the evolution of its spatial heterogeneity.

## Methods

### Design

Population based spatio-temporal study using “hospital service areas” (HSAs) as a unit of analysis.

### Setting

The study was conducted in the sNHS, a decentralized structure of 17 regional National Health Services (NHS) administered by the 17 Autonomous Governments of the Spanish regions [16, 17]. During the study period healthcare coverage was almost universal. Regional NHSs operate an extensive network of hospitals (about 75 % of acute hospital beds in Spain), and specialized outpatient and primary healthcare centres. Healthcare in this network is free of charge (except for co-payment for outpatient prescriptions) and supported by Regional Government budgets. In 2013 the 17 sNHS regions were organized into 203 HSAs, geographical territories –most of them between 150,000 and 250,000 people– served by one NHS hospital that provides specialized inpatient and outpatient care to the residents in its area. Primary care is organized into local zones –most of them between 5000 and 25,000 people– associated to their respective HSA. Due to these organizational characteristics (geographical planning, minimal accessibility barriers, and the practical absence of economic incentives to providers), patients receive most of their inpatient and outpatient care in the HSA where they live.

### Sources of data

The Minimum Basic Hospital Discharge Dataset of the Regional NHSs from 2002 to 2013 was used to search for COPD-PPH admissions. This database provides clinical and sociodemographic information on all hospital discharges in the sNHS, including diagnoses and procedures coded according to the International Classification of Diseases 9th revision Clinical Modification (ICD9CM). The population denominators for each year were obtained from the annual census of the Spanish National Institute of Statistics.

### Population

All 2002 to 2013 hospital admissions of patients aged 20 and over for a COPD-PPH condition defined as following the Spanish validation [18, 19] of the US Agency for Healthcare Research and Quality Prevention Quality Indicators [2]. This Spanish version is similar to the US version (see Additional file 1, e-Appendix 1 for differences between versions) but with some ICD9CM codes adapted to the most common codification patterns in the sNHS and have been used in previous studies [5, 20, 21].

### Main endpoint

Age-sex standardized rates of COPD-PPH by 10,000 persons-years (men, women and total) for each HSA.

### Analysis

First, all 2002 to 2013 COPD-PPH admissions of patients aged 20 and over were selected, linked to their HSA of residence and aggregated into 14 age-sex groups (20–44, 45–64, 65–69, 70–74, 75–79, 80–84, 85 and over, for both genders). Age-sex standardized rates were calculated by applying age-sex specific weights representing the importance of each group in the overall population of each area. Variation among HSAs was assessed through the ratio between the 95^th^ and 5^th^ percentiles, the ratio between the 75^th^ and 25^th^ percentiles and the coefficient of variation of the COPD-PPH standardized rates per 10,000 persons-year. Under the hypothesis that risk remains constant in space and time, the expected number of cases per HSA was estimated by applying the rate for all areas over the 12 years to the population at risk in each HSA in the respective year. Standardized Hospitalization Ratios (SHRs) were estimated using the ratio of observed-to-expected cases, interpretable as the maximum estimate of the risk ratio of admissions for a COPD-PPH in that area in that period.

We use a Bayesian hierarchical approach to model the different risk structures of dependence in both space and time. In the first level of this hierarchical modelization, we assume that, conditional on the underlying relative risk, the number of counts *y*_*jt*_ in the *j th* area at the *t th* time period follows a Poisson distribution with mean *u*_*jt*_ *= e*_*jt*_*r*_*jt*_, where *e*_*jt*_ is the number of expected counts and *r*_*jt*_ the unknown relative risk. In the second level, the *log(r*_*ij*_*)* was expressed as the sum of the components representing the individual and independent contributions to the risk in a specific HSA and period [*log(r*_*ij*_*) = intercept + S*_*i*_ *+ T*_*j*_ *+ ST*_*ij*_]*,* where the intercept term gives the initial level of risk that is shared by all regions and periods. The main effects *S*_*i*_ and *T*_*j*_ represent the additional risk of living in region *i* and period *j* and the second order interaction term *ST*_*ij*_ represent the risk contribution due to a combination of the effects that cannot be explained additively by the main effects. In the third level, a hyperparameter-prior distribution was assumed where the spatial effect was modelled following a convolution CAR prior [22]. The temporal main effect was a combination of a time-unstructured (exchangeable) and a time structured effects (first order random walk), and the interaction term can be thought of as the independent unobserved covariates for each combination of region and period (*i,j*), thus without any structure (Type I in the Knorr-Held classification) [23] .

As a summary measure of the uncertainty surrounding the estimate of relative risk, the posterior expected excedence probability (Pr(RR > 1)) is represented [24]. Integrated nested Laplace approximations (INLAs) were used as a tool for Bayesian inference [25]. For this purpose, we used R-INLA with the option of simplified Laplace estimation of the parameters, a package available in the R environment [26].

## Results

**Fig. 1:**
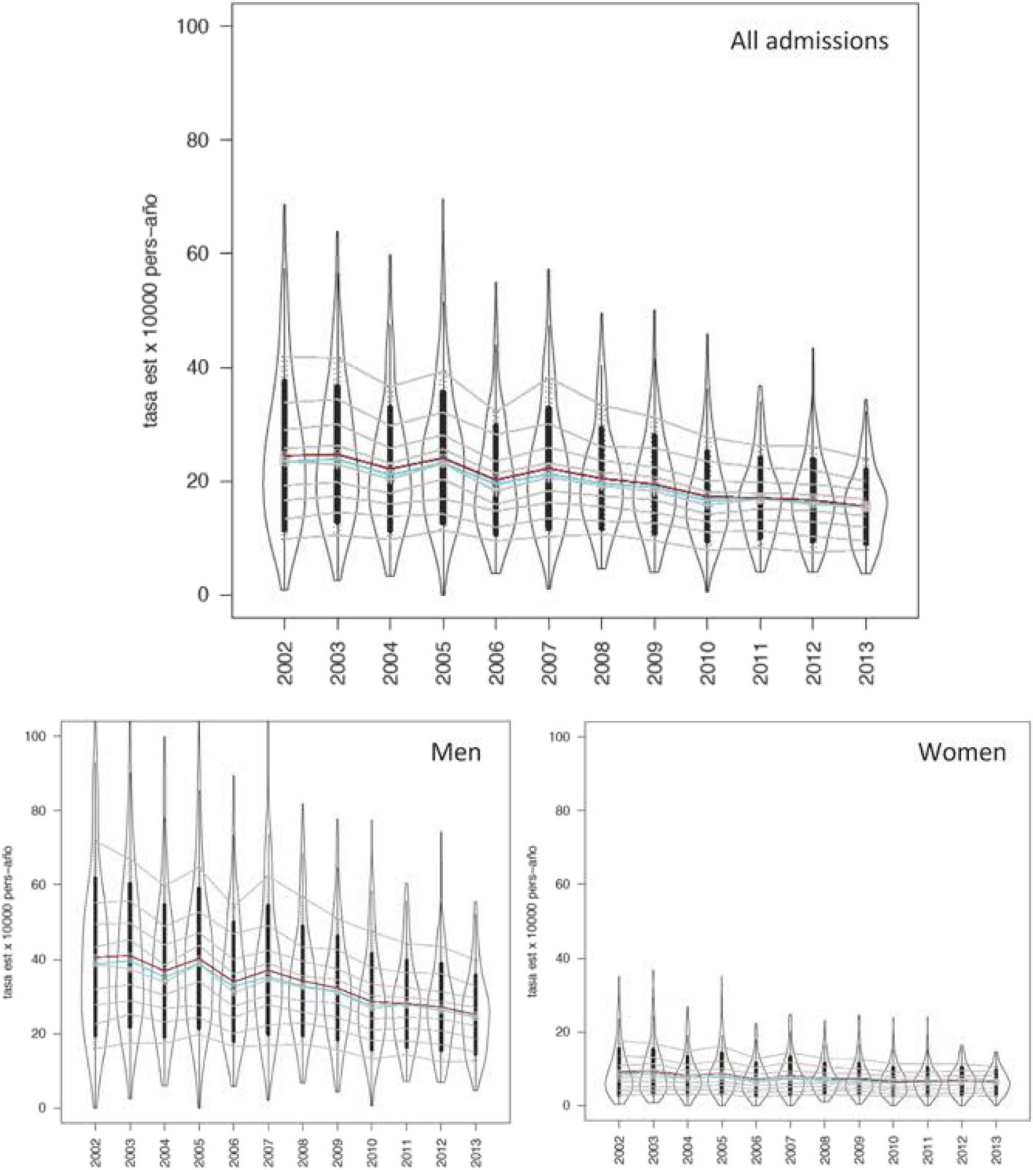
Distribution of COPD-PPH standardized rates among Hospital Service Areas for the overall population, men and women (Spanish NHS, 2002–2013). *Foot:* COPD-PPH: Chronic Obstructive Pulmonary Disease for Potentially Preventable Hospitalizations; NHS: National Health System. Gray lines represent deciles and coloured lines the median (red) and the mean (blue)

COPD-PPH admissions in people aged 20 and over declined from 75,036 to 61,348 during the study period (Table 1, Fig. 1), more markedly in men (from 60,485 to 48,193; −20.32 %) than women (from 14,551 to 13,155; −9.59 %). Women’s hospitalizations increased from 19.4 to 21.5 % (+10.9 %) and the patients’ mean age increased from 73,2 to 74,2 years old at the expense of men (from 72,8 to 74,7 years old vs. a decrease from 74,5 to 72,5 in women). Because the population over 20 rose in this period from 33.3 million in 2002 to nearly 37.7 million in 2009, COPD-PPH age-sex standardized rates declined more sharply: from 24.5 to 15.5 per 10,000 py (−36.7 %; from 40.6 to 25.1, −38.2 % in men, and from 9.1 to 6,4, −29.7 % in women).

**Table 1:** Number of COPD-PPH admissions and standardized rates in people aged 20 and over in the Spanish National Health System (rates by 10,000 person-year; 2002–2013)

Year	2002	2003	2004	2005	2006	2007	2008	2009	2010	2011	2012	2013
Population aged 20+ (n, millions)	33.34	34.13	34.66	35.45	36.09	36.53	37.15	37.49	37.67	37.79	37.86	37.75
Population aged 65+ (%)	21.60	21.41	21.17	20.79	20.80	20.69	20.61	20.84	21.01	21.37	21.67	22.03
Hospitalizations (n)	75 036	77 893	72 276	80 027	68 623	77 195	72 647	70 952	64 583	65 315	64 932	61 348
Men	60 485	63 180	58 642	65 176	55 959	62 813	58 901	57 384	51 981	52 364	51 592	48 193
Women	14 551	14 713	13 634	14 851	12 664	14 382	13 746	13 568	12 602	12 951	13 340	13 155
% Women	19.37	18.91	18.87	18.58	18.48	18.68	18.95	19.13	19.53	19.86	20.57	21.49
Patients’ age (mean, years)	73.17	73.32	73.58	73.94	73.86	74.16	74.25	74.05	73.76	74.26	74.60	74.21
Men	72.84	73.00	73.34	73.76	73.84	74.19	74.27	74.24	73.95	74.51	74.94	74.68
Women	74.53	74.66	74.61	74.76	73.94	74.03	74.16	73.22	72.99	73.26	73.28	72.50
Standardized Rate 10,000 p-y	24.46	24.70	22.13	24.05	20.19	22.24	20.45	19.42	17.26	17.03	16.64	15.53
Men	40.62	41.03	36.89	40.21	33.97	37.11	34.20	32.29	28.60	28.07	27.19	25.11
Women	9.07	9.13	8.07	8.65	7.05	8.06	7.35	7.16	6.45	6.50	6.59	6.40
Relative Risk of admission	1.19	1.22	1.11	1.19	1.02	1.11	1.03	0.98	0.86	0.85	0.83	0.77

Relative Risk in relation to the whole 2002–2013 hospitalization rate

*COPD-PPH* Chronic Obstructive Pulmonary Disease for Potentially Preventable Hospitalizations*, p-y* persons-years

**Fig. 2:**
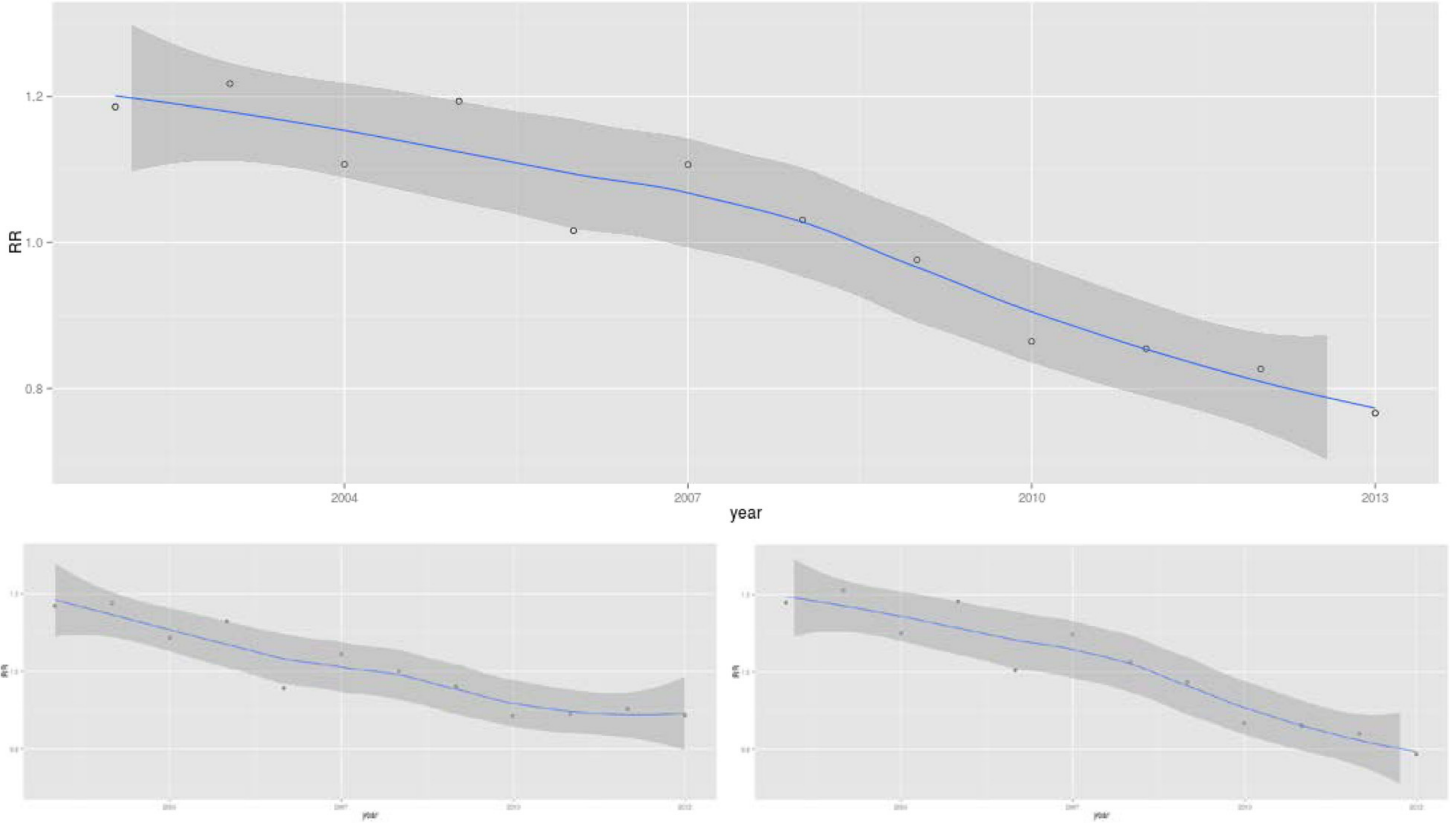
Age-sex adjusted relative risk of being hospitalized for a COPD-PPH condition for the overall population, men and women (Spanish NHS, 2002–2013). *Foot:* COPD-PPH: Chronic Obstructive Pulmonary Disease for Potentially Preventable Hospitalizations; NHS: National Health System

**Fig. 3:**
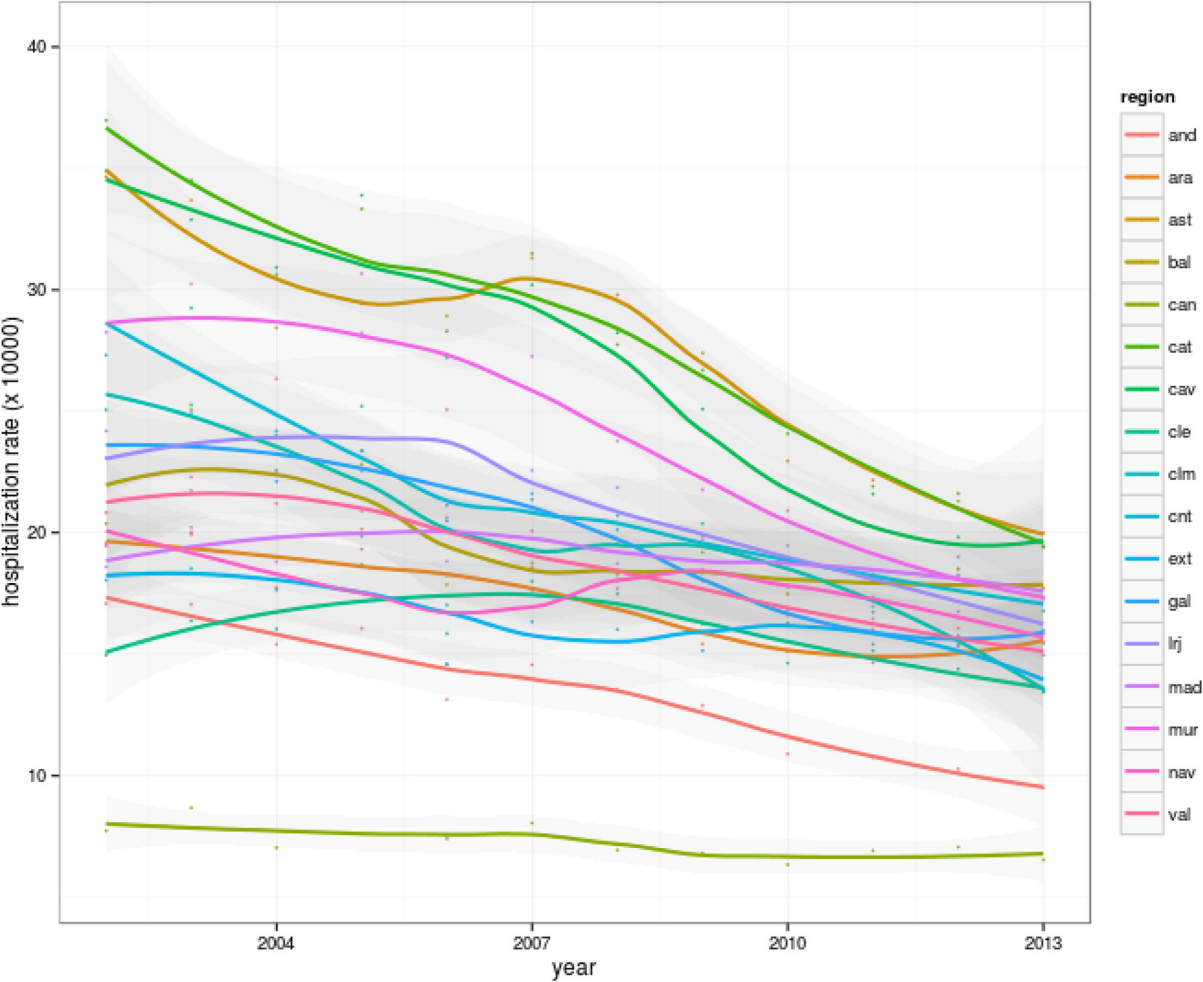
Evolution of COPD-PPH standardized rates by Autonomous Communities (Spanish NHS, 2002–2013). *Foot:* COPD-PPH: Chronic Obstructive Pulmonary Disease for Potentially Preventable Hospitalizations; NHS: National Health System

Figure 2 displays the age-sex adjusted relative risk of being hospitalized for a COPD-PPH condition throughout the study period compared with the average rate over the 12 years studied. This RR, clearly descending throughout almost the whole period, ranged from 1.19 (19 % above the 2002–2013 average) in 2002 to 0.77 (30 % below average) in 2013, but for women the downward trend flattens out in the final years. Both the starting point and the slope were different for the different regions (Fig. 3), with a strong reduction in variability during the study period. For specific trends for Autonomous Regions, gender and age groups see Additional file 2, e-Appendix 2.

Regarding variation among HSAs (Table 2), the standardized rate for the HSA in the P95 was 6.7 times higher than the HSA in the P5 in 2002, while in 2013 this difference was reduced to 4.6 times higher. The ratio between HSAs in the P75 and P25 and the coefficient of variation confirms the variability in descent among HSAs. Violin graphs (Fig. 1) also visually show this double phenomenon of both strong decline and convergence in admission rates among HSAs.

**Table 2:** Variation in standardized rates of COPD-PPH admissions in people aged 20 and over in the Spanish National Health System (2002–2013)

Year	2002	2003	2004	2005	2006	2007	2008	2009	2010	2011	2012	2013
All												
Rate 10000 p-y, HSA in the 10^th^ Percentile	9.82	10.47	9.70	11.36	9.45	10.23	10.69	9.49	7.84	8.21	7.44	7.89
Rate 10000 p-y, HSA in the 90^th^ Percentile	41.94	41.62	36.59	39.33	32.16	38.16	33.37	31.19	27.74	26.24	26.15	23.95
Ratio between 95^th^/5^th^ Percentiles	6.86	4.97	5.57	5.07	4.66	4.75	4.52	5.11	4.71	4.48	4.21	4.62
Ratio between 75^th^/25^th^ Percentiles	2.11	2.00	1.89	1.93	1.85	1.91	1.69	1.79	1.82	1.74	1.77	1.79
Coefficient of Variation	0.53	0.48	0.48	0.47	0.47	0.47	0.43	0.43	0.45	0.41	0.42	0.41
Men												
Rate 10000 p-y, HSA in the 10^th^ Percentile	15.98	17.45	17.37	19.75	16.52	16.80	17.14	15.68	13.14	14.28	12.35	12.58
Rate 10000 p-y, HSA in the 90^th^ Percentile	71.83	62.93	59.70	64.68	54.16	62.33	56.67	50.93	47.41	44.30	43.55	39.84
Ratio between 95^th^/5^th^ Percentiles	6.61	4.85	5.38	4.61	4.46	5.00	4.33	5.36	4.74	4.60	4.48	4.81
Ratio between 75^th^/25^th^ Percentiles	2.07	1.90	1.81	1.90	1.84	1.74	1.73	1.72	1.76	1.72	1.72	1.81
Coefficient of Variation	0.52	0.47	0.48	0.47	0.47	0.47	0.43	0.43	0.45	0.41	0.43	0.42
Women												
Rate 10000 p-y, HSA in the 10^th^ Percentile	2.84	2.98	3.06	3.10	2.26	2.76	2.86	2.84	2.37	2.50	2.53	2.59
Rate 10000 p-y, HSA in the 90^th^ Percentile	17.56	16.82	14.74	16.19	12.68	14.37	12.78	12.67	11.24	11.20	11.26	10.45
Ratio between 95^th^/5^th^ Percentiles	10.75	9.23	9.57	7.74	9.34	9.73	6.02	7.53	7.81	6.90	7.16	6.11
Ratio between 75^th^/25^th^ Percentiles	2.65	2.75	2.27	2.13	2.39	2.43	2.02	2.13	2.20	2.17	2.19	2.04
Coefficient of Variation	0.69	0.66	0.62	0.63	0.62	0.63	0.54	0.58	0.58	0.55	0.53	0.49

*COPD-PPH* Chronic Obstructive Pulmonary Disease admissions for Potentially Preventable Hospitalizations, *HSA* Health Service Areas, *p-y* persons-years

**Fig. 4:**
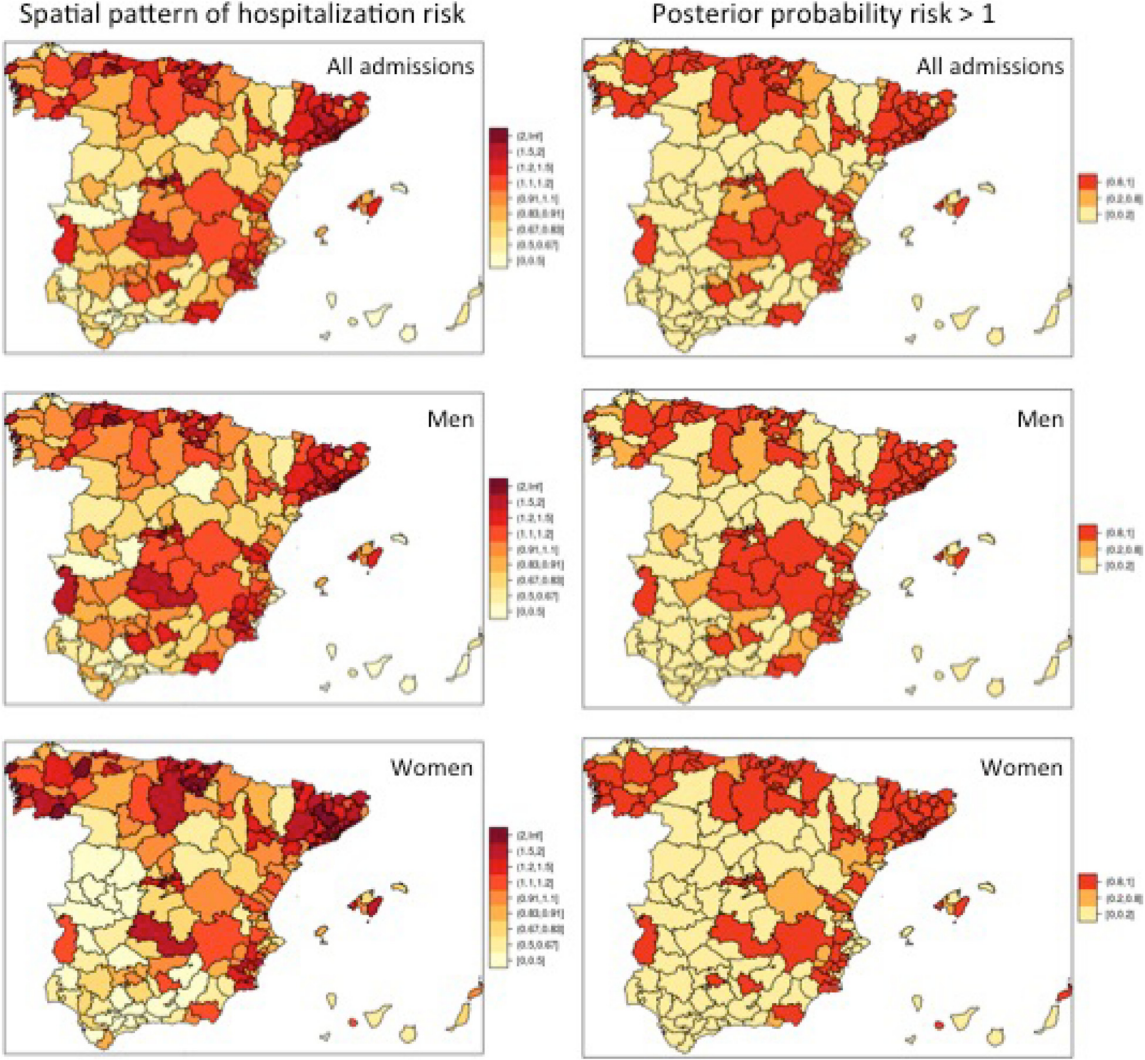
Spatial patterns in standardized rates of COPD-PPH by Health Service Areas for the entire population, men and women (Spanish NHS, 2002–2013). *Foot:* COPD-PPH: Chronic Obstructive Pulmonary Disease for Potentially Preventable Hospitalizations; NHS: National Health System. The left-hand maps show the COPD-PPH admission spatial risk associated to each Health Service Areas to be constant along the period. The right-hand map shows the posterior probability that this spatial risk will be higher than 1 (probabilities above 0.8 indicate high-risk areas)

Figure 4 displays the spatial patterns in standardized rates of COPD-PPH for the entire, male and female population. The left-hand maps show the COPD-PPH admission spatial risk associated to each HAS to be constant along the period. The right-hand map shows the posterior probability that this spatial risk will be higher than 1 (probabilities above 0.8 indicate high-risk HSAs), which seems to concentrate on the Northern coast, the Mediterranean coast and the central eastern area of Spain. See Additional file 3, e-Appendix 3 for the spatio-temporal evolution of COPD-PPH risks for each HSA.

## Discussion

Our study primarily shows a large decline in the rate of admissions for COPD-PPH during the study period. This descending trend is consistent with other work in Spain restricted to hospitalization for COPD exacerbations during the period 2006–2010 [27], but studies in the USA (2001–2012) do not show changes in COPD admission or emergency room visits [28] and in other countries like France (1998–2007) rates have even increased [29]. Other studies in Brazil (1998–2008) [30], Finland (1998–2007) [31], and Australia (1993–2003) [32] show declining hospitalization rates, but sometimes only for men and less pronounced than those found in this study.

Possible causes of this sharp decline are probably diverse and include changes in the therapeutical management of COPD (increases in the utilization of inhaled long-acting beta-agonists, long-acting muscarinic antagonists, and inhaled corticosteroids), improvements in influenza vaccination coverage for high-risk patients, and organizational changes in the hospital accident and emergency departments reducing the volume of emergency hospital admissions for chronic exacerbations (introduction of observation units and higher coordination with Hospital at Home Units and Long Term Care Centres) [33]. Also, Primary Care Centres and Respiratory Medicine Departments in some HSAs have initiated disease management or case management programs for several chronic conditions, including COPD. [34] In this period Spain passed two smoking laws (2006, 2011) banning tobacco in workplaces and other public spaces. Furthermore, the severe economic crisis entailed a significant reduction in family income and a notable increase in excise duties on tobacco, resulting in a marked decline in cigarette consumption that perhaps affected patients with established COPD (on average, of lower socioeconomic strata) more heavily.

All these policies were active in Spain at the end of the study period but they started at different times, in some cases with a defined starting point, in other incrementally over time. Some policies (e.g., changes in admission criteria) would have a direct effect on admission rates, while other (e.g., anti-smoking laws) would have to be mediated through a reduction in the number of smokers or in the average tobacco consumption. We think that, on a downward trend derived from better management of COPD and a secular reduction in the tobacco consumption in adult males, changes in hospital admission criteria have been the main determinant of the reduction in COPD admission rates, while the impact of smoking laws could be gradually visible in the near future. But our data do not allow an estimation of the impact, if any, of each one of these cumulative number of different policies on COPD-PPH rates.

Regarding geographical variation among HSAs, this also experienced a meaningful decrease by compression of the highest rates, but significant variability still remains. For example, with the 2013 rates in the HSA on the 90th percentile, the sNHS would have made around 90,000 COPD-PPH hospitalizations that year, compared to only 30,000 with the rates of the HSA on the 10th percentile. The regions with the highest rates of hospitalization (Catalonia, Asturias, Basque country) experienced the largest reductions. Interestingly, Andalusia, coming from one of the lowest rates of hospitalization, also experienced a significant reduction.

Among the study limitations, it is first worth noting that hospitalizations in private hospitals were not included, reducing overall hospitalization rates. The importance of this bias is difficult to quantify because most private Spanish hospitals specialize in elective surgery and deliveries than in chronic conditions, but according to the Spanish Hospital Morbidity Survey 21 % of all hospitalizations for COPD in 2013 were carried out in private hospitals [35]. Both PPH and private provision of health care are associated with socio-economic level, and the private sector serves most of the employees of the Spanish Central Government (not employees of the regional Governments) that are concentrated in certain HSAs (provincial capitals and the Region of Madrid). Second, hospital admission rates, even standardized by age and sex, do not fully account for the differences in disease prevalence between areas or in the distribution of particularly vulnerable subpopulations [36, 37]. Third, the use of a Spanish PPH definition [18, 19] increases the internal study validity, but limits the contrast of our results with studies that used other definitions.

## Conclusions

PPH have been adopted (and adapted) by different national and international organizations [38–40] and are currently a common and increasingly used instrument for the evaluation of health care [41, 42]. In Europe, where insurance tends to be universal and primary care is well developed, PPH are interpreted not only as a measure of outpatient care quality, but also of hospitals role in the control of chronic patients (discharging patients who are more or less stable, deciding which patients are admitted, etc.) and proper coordination between different levels of care [43].

Our study shows that COPD-PPH conditions in Spain have undergone a strong decline and a reduction in geographical variation by compression of the highest rates in the last 12 years, suggesting a general improvement in the management of COPD over time, specially in the HSAs with initial higher admission rates. Causes for this improvement and the relative value of each one will require further study, but may be of interest to the development of practical policies in Spain and other countries. Nonetheless, the remaining variability and the behaviour of some regions that have achieved substantial declines suggests that there is still considerable room for improvement.

## Abbreviations

COPD, chronic obstructive pulmonary disease; HSA, hospital service areas; ICD9CM, International Classification of Diseases 9th revision Clinical Modification; NHS, National Health Service; PPH, potentially preventable hospitalizations; RR, relative risk; sNHS, Spanish National Health System

## Additional files

Additional file 1: CODES USED FOR IDENTIFICATION OF PPH-COPD ADMISSIONS. Data description: description of the criteria (including ICD-9-CM codes) for inclusion or exclusion of admissions as PPH-COPD. (PDF 58 kb) Additional file 2: TEMPORAL TRENDS BY AGE-GROUP, SEX AND REGION. Data description: e-Figure 1 and e-Figure 2 show the smoothed trends of PPH-CODP admissions stratified by four age groups and by autonomous regions, for men and women respectively. (PDF 9601 kb) Additional file 3: SPATIO-TEMPORAL EVOLUTION OF THE COPD HOSPITALIZATION RISKS. Data description: e-Figure 1 displays the spatiotemporal evolution of COPD-PPH hospitalization relative risk for each Health Service area compared to the Spain average over the whole period, and e-Figure 2 the posterior probabilities that these relative risks were higher than 1. (PDF 6214 kb)
