# Supplementary material for: Trends and area variations in Potentially Preventable Admissions for COPD in Spain (2002–2013): a significant decline and convergence between areas
Source: BMC Health Serv Res. 2016 Aug 9;16:367. doi: 10.1186/s12913-016-1624-y (PMC4979149; doi:10.1186/s12913-016-1624-y)
Supplement: Additional file 1: — CODES USED FOR IDENTIFICATION OF PPH-COPD ADMISSIONS. Data description: description of the criteria (including ICD-9-CM codes) for inclusion or exclusion of admissions as PPH-COPD. (PDF 58 kb) [file 12913_2016_1624_MOESM1_ESM.pdf]

Trends and area variations in Potentially Preventable Admissions for COPD in Spain (2002-2013): a significant decline and convergence between areas.

Julián Librero, MD, PhD, **Berta Ibañez-Beroiz**, MSc, PhD, **Salvador Peiró**, MD, PhD, **Manuel Ridao-López** , BEcon, Msc, **Clara L. Rodíguez-Bernal**, DMD, PhD, **Francisco J. Gómez-Romero**, MD, **Enrique Bernal-Delgado**, MD, PhD, and the **Spanish Atlas of Medical Practice Variation Research Group**.

Additional file 1

e-APPENDIX 1. CODES USED FOR IDENTIFICATION OF PPH-COPD ADMISSIONS

The PPH-COPD indicator was constructed as admissions in patients aged 20 and older with a primary diagnosis of COPD or a primary diagnosis of a related lung disease accompanied by a secondary diagnosis of COPD. Obstetric admissions and admissions for congestive heart failure, cystic fibrosis, and mental disorders were excluded.

Cases were included if:

Age ≥ **18** years
AND
PRIMARY DIAGNOSIS = **491.1** OR **491.20** OR **491.21** OR **491.22** OR **491.8** OR **491.9** (Chronic bronchitis) OR **492.0** OR **492.8** (Emphysema) OR **493.20** OR **493.21** OR **493.22** (Asthma) OR **494.0** OR **494.1** (Bronchiectasis) OR **496*** (Chronic airway obstruction, not classified elsewhere)
OR
PRIMARY DIAGNOSIS = **466.0** (Acute bronchitis and bronchiolitis) OR **490** (Bronchitis, not specified as acute or chronic) AND THERE IS A SECONDARY DIAGNOSIS = **491*** OR **492*** OR **493*** OR **494*** OR **496***
OR
PRIMARY DIAGNOSIS = **518.81** OR **518.84** (respiratory failure) AND THERE IS A SECONDARY DIAGNOSIS = **491.21** OR **491.22** OR **493.21** OR **493.22** OR **494.0** OR **494.1**

Cases were excluded if

THERE IS A SECONDARY DIAGNOSIS = **630** *to* **677** (obstetrical care) OR **428** OR **402.01** OR **402.11** OR **402.91** OR **404.01** OR **404.03** OR **404.11** OR **404.13** OR **404.91** OR **404.93** OR **398.91** (congestive heart failure**) OR 277.0** OR **747.21** OR **748.3** OR **748.4** OR **748.5** OR **748.6*** OR **748.8** OR **748.9** OR **750.3** OR **759.3** OR **770.7** OR **747.31** OR **747.32** OR **747.39** (Cystic fibrosis) OR **295.0** *to* **295.9** OR **296.0** *to* **296.99** OR **297.0** *to* **297.9** OR **298.0** *to* **298.9** OR **300.0** *to* **300.9** OR **301.0** *to* **301.9** OR **303.91** OR **304.01** OR **304.11** OR **304.21** OR **304.31** OR **304.41** OR **304.51** (Mental disorders)

*Indicates that all 4th or 5th digit codes following the respective previous code were included.
