# Supplementary material for: Trends and area variations in Potentially Preventable Admissions for COPD in Spain (2002–2013): a significant decline and convergence between areas
Source: BMC Health Serv Res. 2016 Aug 9;16:367. doi: 10.1186/s12913-016-1624-y (PMC4979149; doi:10.1186/s12913-016-1624-y)

Trends and area variations in Potentially Preventable Admissions for COPD in Spain (2002-2013): a significant decline and convergence between areas.

Julián Librero, MD, PhD, **Berta Ibañez-Beroiz**, MSc, PhD, **Salvador Peiró**, MD, PhD, **Manuel Ridao-López** , BEcon, Msc, **Clara L. Rodíguez-Bernal**, DMD, PhD, **Francisco J. Gómez-Romero**, MD, **Enrique Bernal-Delgado**, MD, PhD, and the **Spanish Atlas of Medical Practice Variation Research Group**.

Additional file 2

e-Appendix 2. TEMPORAL TRENDS BY AGE-GROUP, SEX AND REGION.

e-Figure 1 shows the smoothed trends of PPH-CODP admissions for men stratified by four age groups and by autonomous regions. All age-groups show declining trends except the 85 years old and over group which shows an ascending trend to the midway of the period and a subsequent decline.

e-Figure 2 shows the smoothed trends of PPH-CODP admissions for women stratified by four age groups and by autonomous regions. While most of the groups maintain the downward trend (not in all regions), the youngest groups show upward trends.

e-Appendix 2, e-Figure 1. Smoothed trends of PPH-CODP rates for men stratified by four age groups and by autonomous region.

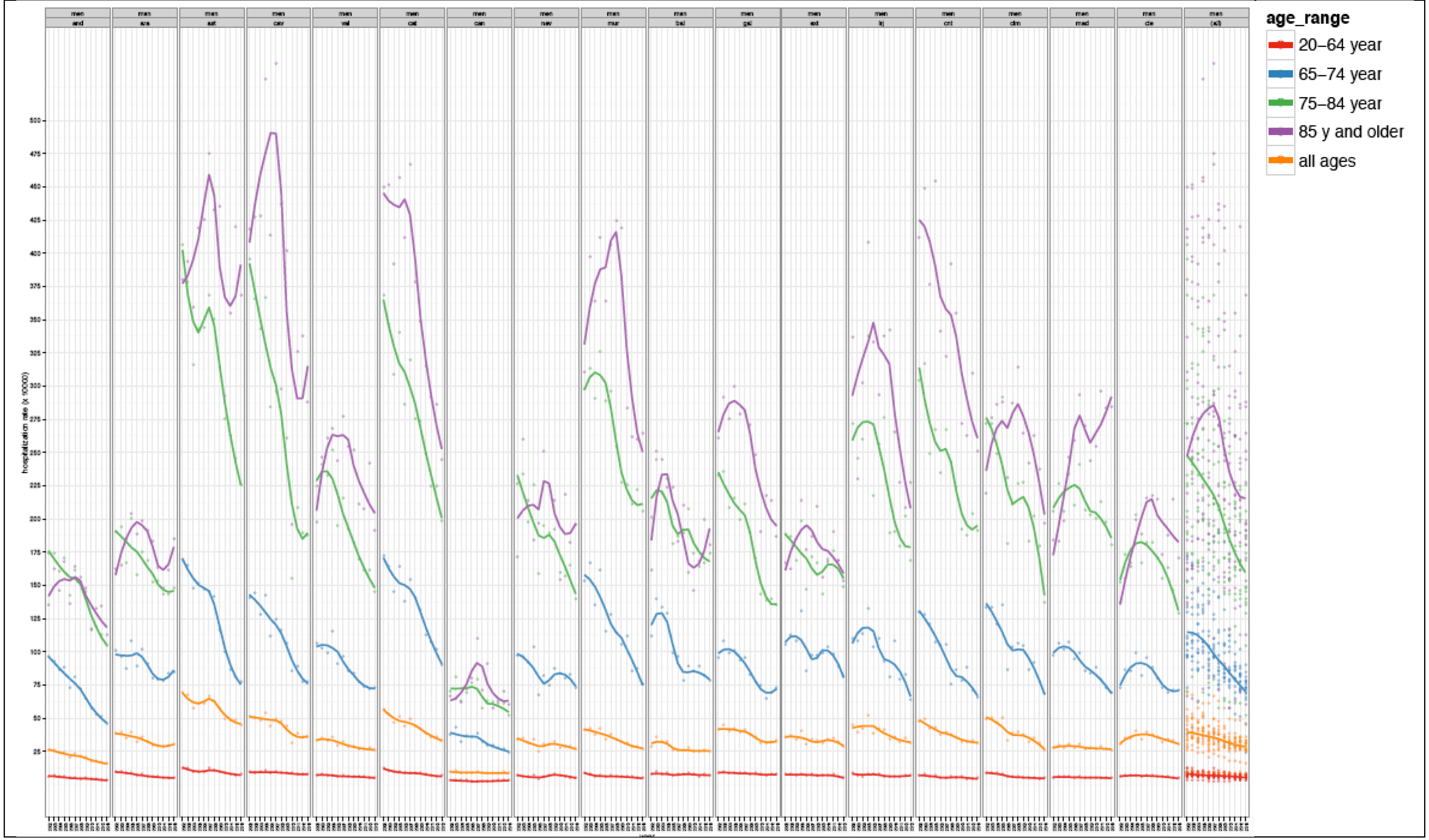

e-Appendix 2, e-Figure 1. Smoothed trends of PPH-CODP rates for women stratified by four age groups and by autonomous region.

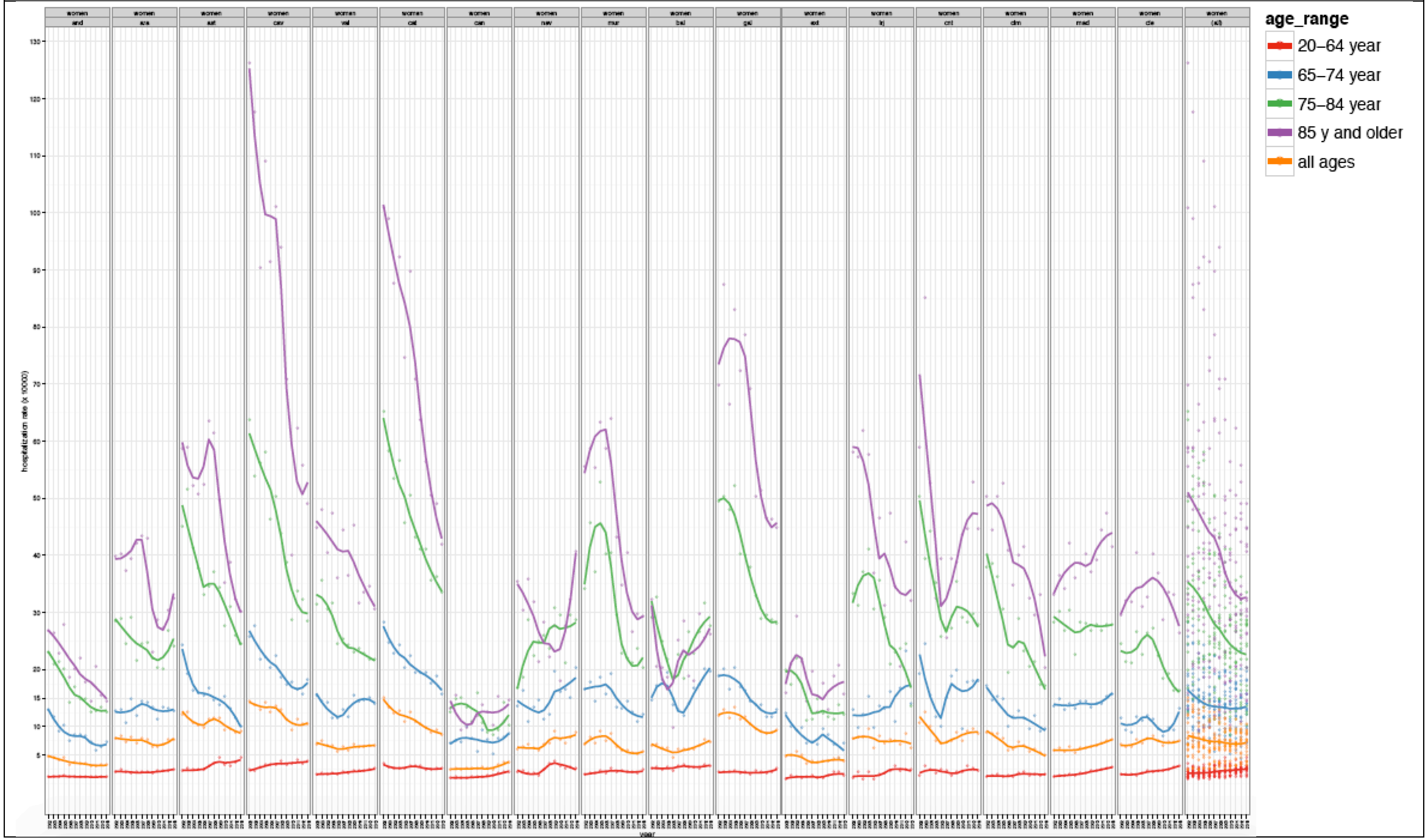

Supplement: Additional file 2: — TEMPORAL TRENDS BY AGE-GROUP, SEX AND REGION. Data description: e-Figure 1 and e-Figure 2 show the smoothed trends of PPH-CODP admissions stratified by four age groups and by autonomous regions, for men and women respectively. (PDF 9601 kb) [file 12913_2016_1624_MOESM2_ESM.pdf]
