# Supplementary material for: Trends and area variations in Potentially Preventable Admissions for COPD in Spain (2002–2013): a significant decline and convergence between areas
Source: BMC Health Serv Res. 2016 Aug 9;16:367. doi: 10.1186/s12913-016-1624-y (PMC4979149; doi:10.1186/s12913-016-1624-y)
Supplement: Additional file 3: — SPATIO-TEMPORAL EVOLUTION OF THE COPD HOSPITALIZATION RISKS. Data description: e-Figure 1 displays the spatiotemporal evolution of COPD-PPH hospitalization relative risk for each Health Service area compared to the Spain average over the whole period, and e-Figure 2 the posterior probabilities that these relative risks were higher than 1. (PDF 6214 kb) [file 12913_2016_1624_MOESM3_ESM.pdf]

Trends and area variations in Potentially Preventable Admissions for COPD in Spain (2002-2013): a significant decline and convergence between areas.

Julián Librero, MD, PhD, **Berta Ibañez-Beroiz**, MSc, PhD, **Salvador Peiró**, MD, PhD, **Manuel Ridao-López** , BEcon, Msc, **Clara L. Rodíguez-Bernal**, DMD, PhD, **Francisco J. Gómez-Romero**, MD, **Enrique Bernal-Delgado**, MD, PhD, and the **Spanish Atlas of Medical Practice Variation Research Group**.

Additional file 3

e-Appendix 3. SPATIO-TEMPORAL EVOLUTION OF THE COPD HOSPITALIZATION RISKS

e-Figure 3 displays the spatio-temporal evolution of COPD-PPH hospitalization risk for each HSA compared to the Spain average over the whole period, and e-Figure 2 the posterior probabilities that these Relative Risks were higher than 1. Combining the information provided for both figures, an increased risk is observed in the first few years on the Atlantic and Mediterranean coasts and on the centre of Spain, but at the end of the period only a few HSAs exhibit significanlyt higher relative risks.

e-Appendix 3, e-Figure 1. Spatio-temporal evolution of COPD-PPH hospitalization risks for each Health Service Area compared to the whole of Spain in the study period (2002-2013).

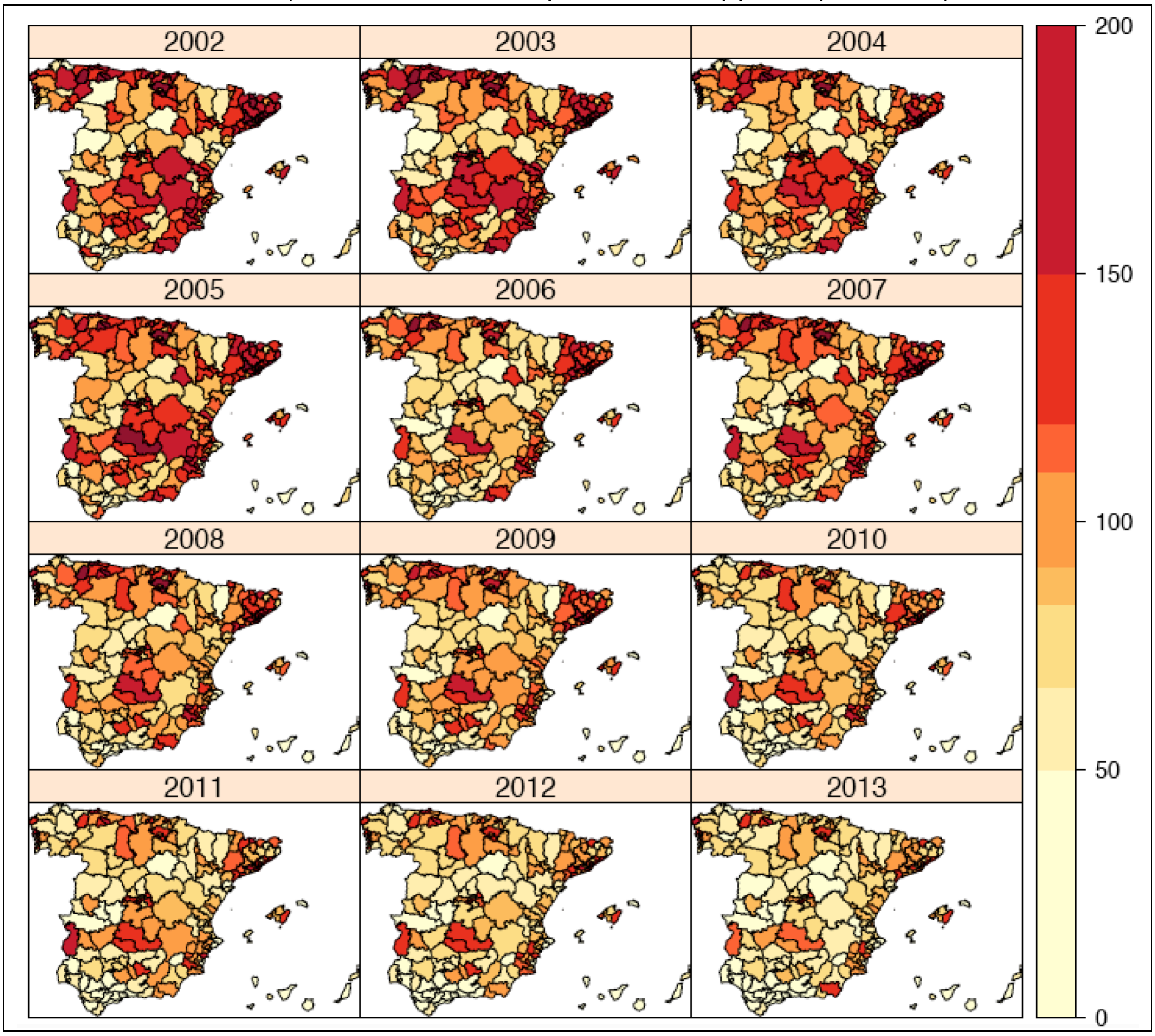

COPD-PPH: Chronic Obstructive Pulmonary Disease for Potentially Preventable Hospitalizations. Colours represent standardized hospitalization ratios (SHR). The average SHR of Spain in the 2002-2013 period is equal to 100 (coloured orange); a SHR of 150 represents a hospitalization risk 50% higher than the average.

e-Appendix 3, e-Figure 2. Spatio-temporal evolution of COPD-PPH hospitalization risks for each Health Service Area. Posterior probabilities for Relative Risk >1.

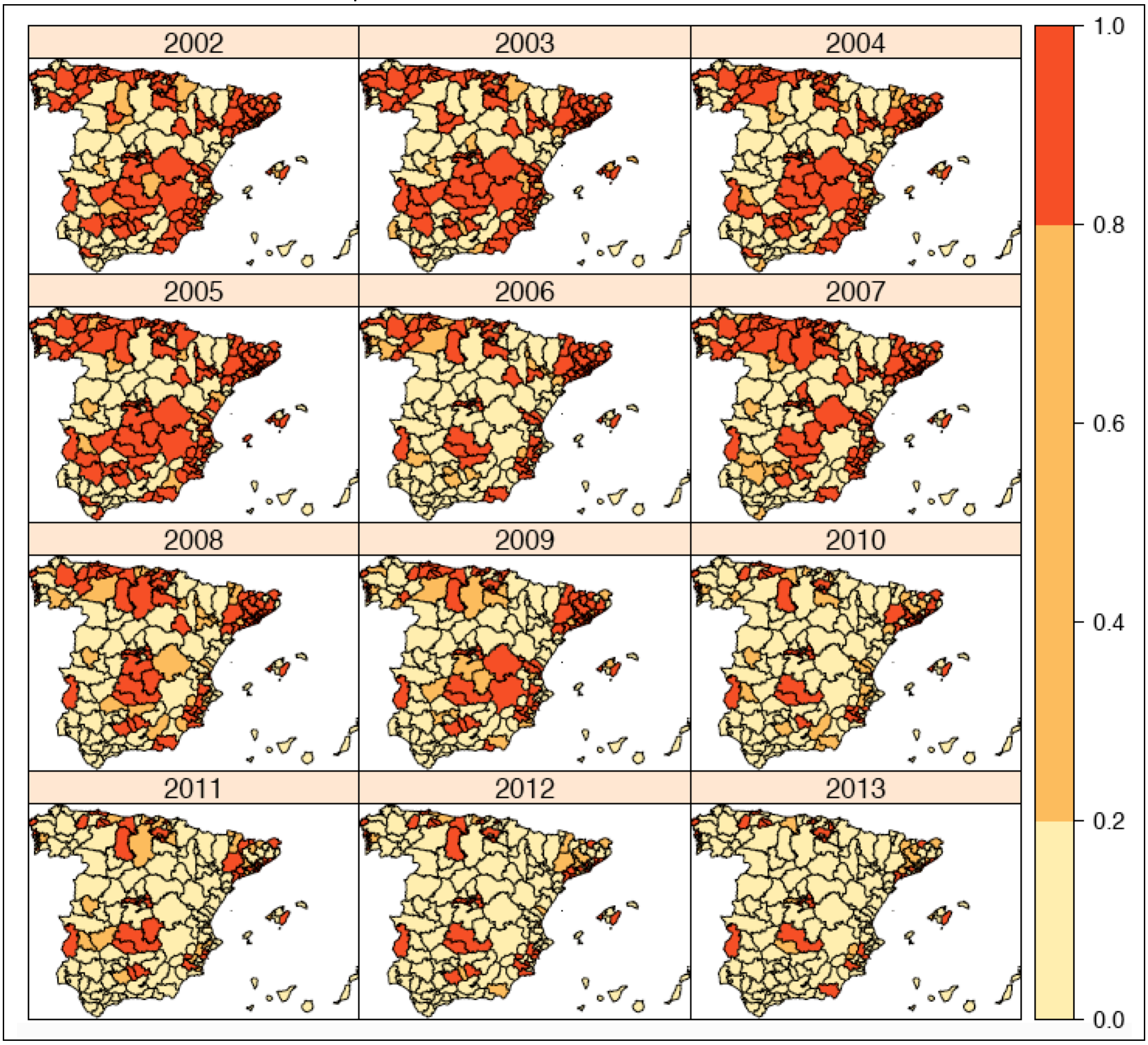

COPD-PPH: Chronic Obstructive Pulmonary Disease for Potentially Preventable Hospitalizations. Dark-brown coloured regions are those for which the probability of having a relative risk of a COPD PPH admission higher than 1 is >0.8, and green ones are areas for which this relative risk is below <0.2
